# EGF通过STAT5调控肺腺癌A549细胞中COX-2的机制

**DOI:** 10.3779/j.issn.1009-3419.2013.04.01

**Published:** 2013-04-20

**Authors:** 守强 曹, 桂彬 赵, 庆 董, 敬泉 韩, 衍忠 辛, 宇博 闫, 吉尧 李, 键 崔

**Affiliations:** 150001 哈尔滨，哈尔滨医科大学附属第四医院胸外科 Department of Thoracic Surgery, the Fourth Affiliated Hospital of Harbin Medical University, Harbin 150001, China

**Keywords:** STAT5, COX-2, 肺肿瘤, A549, EGF, STAT5, COX-2, Lung neoplasms, A549, EGF

## Abstract

**背景与目的:**

已有的研究表明COX-2在肺癌发生发展过程中起关键作用, 它被一些细胞因子和生长因子所诱导产生, 并受到JAK/STAT等信号通路的调控, 抑制COX-2的表达能阻止肺癌的发展。本研究旨在探讨表皮生长因子(epidermal growth factor, EGF)在人肺腺癌A549细胞中对STAT5激活效应, 以及STAT5信号通路对COX-2调控机制。

**方法:**

应用免疫荧光法及Western印迹法检测人肺腺癌A549细胞中EGF对STAT5的激活现象。分别用野生型STAT5(AdWT STAT5), STAT5显性负突变体(AdCMV5 Stat5a△740)以及STAT5 siRNA转染A549细胞, 并用EGF对后两组转染细胞加以刺激, 使STAT5及p-STAT5的表达发生变化, 再用RT-PCR检测A549细胞中的COX-2 mRNA表达。

**结果:**

在体外A549细胞中STAT5无激活; EGF可以诱导STAT5的激活, 促使磷酸化的STAT5穿梭入核; STAT5的激活是EGF诱导COX-2上调表达的必要条件; 非磷酸化的STAT5可能通过非转录激活的途径参与了COX-2表达的调控。

**结论:**

在A549细胞中STAT5可以通过磷酸化和非磷酸化两种途径来实现对COX-2的调控。

## Abstract (en)

**Background and objective:**

It has been proved that cyclooxygenase-2 (COX-2) is a key factor in lung cancer oncogenesis.COX-2 can be induced by a number of cytokines and growth factors and can be regulated by the JAK/STAT signaling pathway.Inhibiting the expression of COX-2 can prevent the development of lung cancer.The aim fo this study is to investigate whether the epidermal growth factor (EGF) can stimulate the signal transducers and activators of transcription 5 (STAT5) as well as to discover the effects of the STAT5 signaling pathway on the COX-2 in human lung adenocarcinoma A549 cells.

**Methods:**

The phenomenon of STAT5 activation stimulated by the EGF was assayed through immunofluorescence and Western blot.The adenovirus containing the wild-type (WT)-STAT5 (AdWT-STAT5) plasmid, dominant-negative (DN)-STAT5 (Ad-CMV5Stat5aΔ740) plasmid, and STAT5 siRNA were transfected into A549 cells.The latter two groups were stimulated using EGF. Reverse transcriptase polymerase chain reaction was used to detect the mRNA expression of COX-2.

**Results:**

STAT5 was not activated in A549 cells *in vitro*.EGF stimulation significantly increased the level of the p-STAT5 protein and induces the shuttling of p-STAT5 from the cytoplasm into the nucleus.STAT5 activation was crucial for the COX-2 expression induced by the EGF.STAT5 was required for COX-2 expression, but can mediated the effects of the COX-2 expression through pathways that were independent of transcriptional activation.

**Conclusion:**

COX-2 expression is dependent on STAT5 phosphorylation.A second pathway does not require STAT5 phosphorylation.

肺癌是导致人类死亡的第一高发癌症。肺腺癌是非小细胞肺癌(non-small cell lung cancer, NSCLC)的一种, 虽然可以通过手术切除和放化疗进行治疗, 但患者预后通常较差。近几年随着小分子靶向药物治疗的兴起, 使部分肺腺癌患者的生存期明显延长, 生活质量得到改善。然而临床研究^[1]^表明, 表皮生长因子受体(epidermal growth factor receptor, EGFR)突变阳性的肺腺癌患者对EGFR酪氨酸激酶抑制剂(tyrosine kinase inhibitors, TKIs)的有效率可达70%-80%, 而野生型的患者有效率仅约10%-20%, 治疗存在局限性。即便EGFR-TKI初始治疗有效的患者, 随着治疗时间的延长, 最终几乎都会出现获得性耐药, 而且, 严重的皮肤副反应等并发症常使很多患者被迫停药。以上种种问题限制了EGFR-TKI的推广使用, 在不久的将来, EGFR-TKI必将被副作用小、适用人群广的靶向药物所取代。

STAT5是STAT家族中的成员, 受细胞因子或生长因子激活后发生磷酸化改变, 由细胞浆穿梭入细胞核, 参与转录调控, 并在细胞的生长及分化过程中起到关键作用。在人体内很多组织存在STAT5的表达, 而且血液、乳腺、头颈部及前列腺肿瘤等存在STAT5的激活, 其参与了肿瘤细胞的增殖、分化及凋亡等生物学行为。COX-2是COX家族中的一员, COX-2在多种实体瘤如肺癌、乳腺癌、前列腺癌、膀胱癌中均存在较高表达, 并参与了肿瘤细胞的调亡、肿瘤侵袭、血管发生及肿瘤转移等过程。越来越多的研究表明COX-2在肺癌发生过程中起关键作用, COX-2的过度表达可以抑制细胞调亡, 刺激血管生成, 促进肿瘤细胞的浸润与转移, 导致肺癌疾病进展^[2]^, 并可作为判断肺癌预后的指标^[3]^。而COX-2抑制剂无论与其它药物联合治疗或单独使用, 均因其对肺癌血管生成的抑制而具有治疗作用^[2]^。因此, COX-2成为肺癌未来诊断与治疗研究的新热点。但在肺腺癌中COX-2是否受到细胞因子的刺激后表达发生改变, 以及STAT5是否参与了COX-2表达的调控等机制至今仍不明确。带着这些疑问, 我们对肺腺癌A549细胞中STAT5对COX2的调控机制进行了研究。

## 1 材料与方法

### 1.1 材料

人肺癌细胞株A549购自中国科学院典型培养物保藏委员会细胞库。STAT5和p-STAT5a/b兔抗人抗体购自美国Santa Cruz公司, FITC标记的羊抗兔二抗购自美国Sigma公司。STAT5短片断干扰RNA(small interfer RNA, siRNA)(ON-TARGETplus SMARTpool siRNA), ON-TARGETplus Non-targeting siRNA(阴性对照)及DharmaFECT siRNA转染试剂均购自美国Dharmacon公司。STAT5显性负突变体(AdCMV5 Stat5a△740)及野生型STAT5(AdWTSTAT5)质粒由名古屋市立大学医学科学院Hiroko Yamashita教授惠赠。ProteoJET^TM^细胞浆和细胞核蛋白提取试剂盒购自加拿大Fermentas公司。蛋白提取试剂盒购自美国Milipore公司。Bradford蛋白定量试剂盒购自美国Pierce公司。含有STAT5结合位点的生物素标记双链探针(5' -biotin-AGATTTCTAGGAATTCGCAG-3' )购自美国Affymetrix公司。DuoSet IC(Intracellular)Active Transcription Factor Assays(用于检测转录因子结合实验)购自英国R & D公司。辣根过氧化物酶(horseradish peroxidase, HRP)标记的羊抗兔抗体购自美国Chemicon公司。Trizol试剂购自Invitrogen公司。RT-PCR试剂盒购自大连TaKaRa公司。PCR所用引物均由大连TaKaRa公司提供。

### 1.2 方法

#### 1.2.1 细胞培养

人肺癌细胞株A549用含有10%胎牛血清、100 μg/mL青霉素、100 μg/mL链霉素的RPMI-1640完全培养基(美国Gibco公司)在37 ℃、5%CO_2_、相对湿度为90%的培养箱中培养。随后将细胞接种于12孔板中进行EGF(Invitrogen公司, 100 ng/mL)激活实验及转染研究。

#### 1.2.2 免疫荧光法检测STAT5核穿梭现象

A549细胞爬片24 h, 待细胞伸展并牢固贴附玻片后用磷酸缓冲盐溶液(phosphate buffer saline, PBS)洗涤细胞, 更换为含0.1%血清的培养基饥饿培养过夜。次日更换为含100 ng/mL EGF和10%胎牛血清的完全培养基, 1 h后, 用PBS冲洗, 4%多聚甲醛固定10 min, 0.25% Triton X-100透化处理细胞, 5%胎牛血清封闭1 h, 用兔抗人STAT5或p-STAT5a/b一抗(1:100稀释)4 ℃孵育过夜。PBS洗涤后, 用FITC标记的羊抗兔二抗(1:200稀释)孵育2 h。Hoechst33258细胞核染色后, 在荧光显微镜下观察并拍照。

#### 1.2.3 STAT5 siRNA的转染及分组研究

待A549细胞铺满板底约80%-90%时, 根据商品说明书使用DharmaFECT siRNA转染试剂对细胞进行STAT5 siRNA转染(50 nM)。ON-TARGETplus Non-targeting siRNA作为阴性对照进行转染, 转染72 h后Western blot法验证沉默效率。并根据研究需要分为6组:未转染组; 转染阴性对照siRNA组; EGF刺激组; 转染阴性对照siRNA加EGF刺激组; 转染STAT5 siRNA加EGF刺激组; 转染STAT5 siRNA组。

#### 1.2.4 STAT5显性负突变体(dominant negative mutant, DN)及野生型STAT5的转染及分组研究

腺病毒介导的Stat5a△740 Stat5a能够阻断STAT5的信号通路的激活^[4]^。待A549细胞铺满板底约70%-80%时, 准备转染质粒。取2支EP管, 于一管中加入10 μg质粒和500 μL无血清、无抗生素的RPMI-1640培养基, 另一管加入20 μg Lipofectamine 2000和500 μL无血清、无抗生素的RPMI-1640培养基。两管混合后, 室温放置30 min, 再加1 mL RPMI-1640培养基, 混匀。吸出孔板中旧培养基, 用无血清、无抗生素的RPMI-1640培养基洗涤一遍, 然后加入已混匀的上述两种混合液。2 h后, 倒掉混合液, 更换为含10%血清的RPMI-1640培养基, 转染72 h后Western blot法验证转染效率。并根据研究需要分为5组:未转染组; 转染野生型STAT5组; EGF刺激组; 转染STAT5显性负突变体加EGF刺激组; 转染STAT5 siRNA加EGF刺激组。

#### 1.2.5 转录因子结合实验

将干预后细胞分为如下5组:未转染组; 转染野生型STAT5组; EGF刺激组; 转染STAT5显性负突变体加EGF刺激组; 转染STAT5 siRNA加EGF刺激组。根据试剂盒说明书提取各组细胞核蛋白。使用Bradford蛋白定量试剂盒进行蛋白质浓度测定。根据文献^[5]^指导进行操作。一抗为兔抗人p-STAT5(100 μL/1:100), 二抗为HRP标记的羊抗兔抗体(100 μL/ 1:200)。

#### 1.2.6 Western blot实验

细胞经上述处理后, 根据试剂盒说明书分别提取细胞核蛋白, 细胞浆蛋白和总蛋白, 首先进行蛋白定量, 从每组中取50 μg蛋白提取物进行SDS聚丙烯酰胺凝胶电泳, 转膜, 以5%脱脂奶粉PBS/0.1% Tween20封闭30 min, 加入1:1, 000倍稀释的一抗(STAT5或p-STAT5), 以β-actin或Histone H1为内参, 4 ℃孵育过夜。PBS/0.1% Tween20洗膜后加入二抗室温孵育2 h。PBS/0.1% Tween20洗膜后ECL发光, 用Chemi-genius凝胶成像系统分析目的蛋白的表达量。

#### 1.2.7 半定量RT-PCR检测

细胞经过上述处理后, 收集细胞, 用Trizol试剂一步法提取细胞总RNA。取总RNA 500 ng进行cDNA第一链合成, 根据RT-PCR试剂盒说明书合成模板。引物序列如下:COX-2(forward:5’-TTCAAATGAGATTGTGGGAAAATTGCT-3' ;reverse:5' -AGATCATCTCTGCCTGAGTATCTT-3' ), β-actin(forward:5’-AAATCGTGCGTGACATTAA-3’; reverse:5’-CTCGTCATACTCCTGCTTG-3’)。PCR体系在94 ℃下变性2 min, 循环条件为94 ℃、30 s, 55 ℃、30 s, 72 ℃、30 s, 共25个循环, 最后延伸10 min。取产物5 μL通过电泳利用琼脂糖凝胶进行分离, 并在紫外线灯下照相。最后通过凝胶灰度分析仪进行统计分析。

### 1.3 统计分析

利用SPSS 13.0软件进行统计学处理, 统计学数据用Mean±SD表示, 多组间均数比较采用单因素方差分析。*P* < 0.05为差异有统计学意义。

## 2 结果

### 2.1 EGF对肺腺癌A549细胞中STAT5活化的影响

为明确在体外A549细胞中是否存在STAT5的激活, 以及EGF是否可以促进STAT5的活化, 我们用EGF对细胞刺激后进行了免疫荧光染色。在荧光显微镜下, 我们发现在部分未经处理的A549细胞中活化的STAT5(p-STAT5)主要位于细胞浆, 且处于极低的激活水平, 而在部分细胞则无p-STAT5的表达。EGF刺激A549细胞后STAT5部分活化穿梭入细胞核, 而且呈高水平激活(图 1)。在未经处理的A549细胞中STAT5位于细胞浆内, 呈高水平表达。EGF刺激A549细胞后细胞浆内STAT5的表达无明显改变(图 2)。经Western blot分析, 结果显示, 在未经处理的A549细胞核中无p-STAT5的表达, 而经EGF激活后, 细胞核中的p-STAT5呈高表达(图 3A)。在EGF刺激A549细胞前后, 细胞浆内的STAT5均呈高表达, 且无明显差异(图 3B)。

**1:**
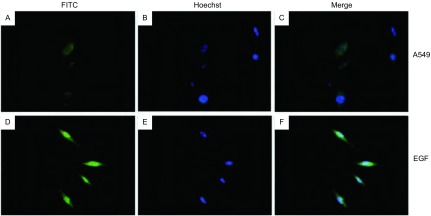
A549细胞和EGF刺激A549细胞后p-STAT5的免疫荧光染色分析。A:p-STAT5在A549细胞中的表达; B:A549细胞的核染色; C:A与B的重叠图像; D:EGF刺激后p-STAT5在A549细胞中的表达; E:A549细胞的核染色; F:D与E的重叠图像。 Immunofluorescence of p-STAT5 in resting and EGF-stimulated human lung adenocarcinoma A549 cells.Upper panels, no EGF stimulation; lower panels, EGF stimulation.(A) and (D), p-STAT5 staining; (B) and (E), Hoechst33258 staining of nuclei; (C) and (F), merged images.

**2:**
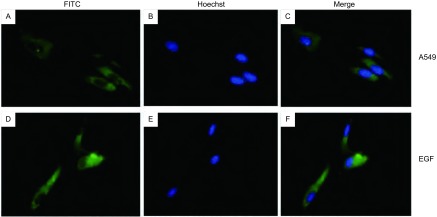
A549细胞和EGF刺激A549细胞后STAT5的免疫荧光染色分析。A:STAT5在A549细胞中的表达; B:A549细胞的核染色; C:A与B的重叠图像; D:EGF刺激后STAT5在A549细胞中的表达; E:A549细胞的核染色; F:D与E的重叠图像。 Immunofluorescence of STAT5 in resting and EGF-stimulated human lung adenocarcinoma A549 cells.Upper panels, no EGF stimulation; lower panels, EGF stimulation.(A) and (D), STAT5 staining; (B) and (E), Hoechst33258 staining of nuclei; (C) and (F), merged images.

**3:**
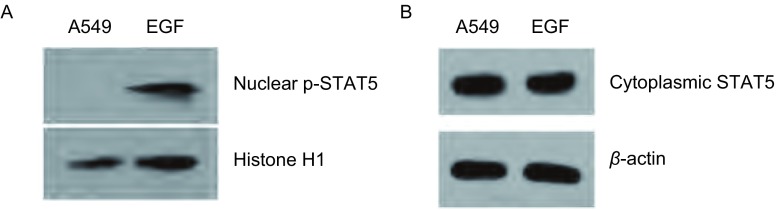
EGF刺激对A549细胞核p-STAT5(A)和细胞浆STAT5(B)表达影响的免疫印迹分析 P-STAT5 (A) and STAT5(B) expression induced by EGF in human lung adenocarcinoma A549 cells.Western blot analysis of nucleus extracts from EGF-stimulated and resting A549 human lung adenocarcinoma cells showing upregulation of nucleus p-STAT5 (A) and no significant of cytoplasmic STAT5 (B).

### 2.2 STAT5 siRNA对A549细胞中STAT5蛋白表达的影响及对COX-2 mRNA表达的影响

Western blot检测结果表明, 与未转染及转染阴性对照siRNA的细胞相比, 转染STAT5 siRNA细胞中STAT5蛋白表达水平明显降低(*P* < 0.05), 提示STAT5 siRNA能明显阻断STAT5的表达, 而EGF刺激后对STAT5的表达无明显影响(图 4A)。另一方面, 与未转染及转染阴性对照siRNA的细胞相比, 转染STAT5 siRNA细胞中COX-2 mRNA的表达明显降低(*P* < 0.05)。EGF虽然可以使COX-2 mRNA表达明显升高, 但在转染STAT5 siRNA细胞中, 此作用被明显削减(*P* < 0.05, 图 4B)。

**4:**
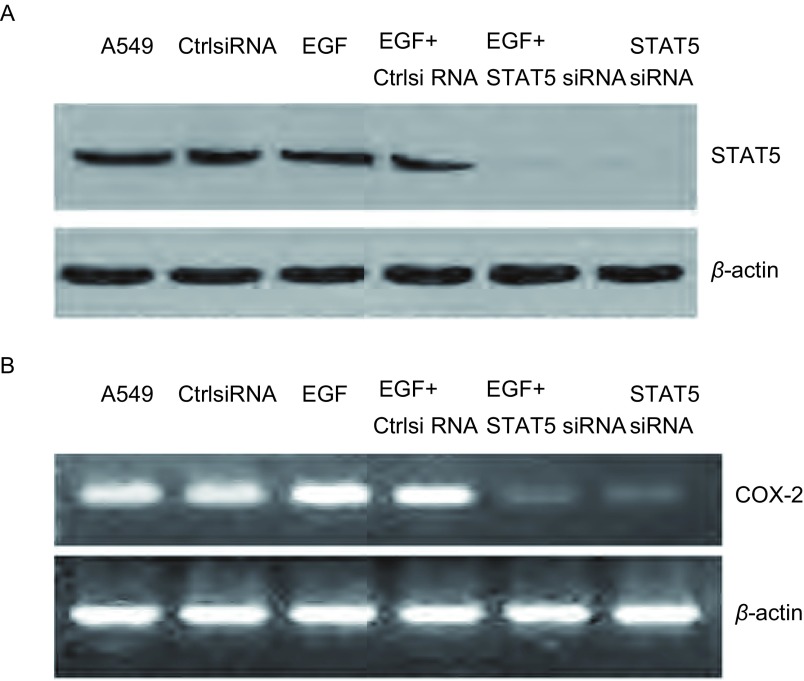
A549细胞转染STAT5 siRNA后以及受到EGF刺激后对STAT5表达影响的免疫印迹分析(A)和COX-2 mRNA表达影响的电泳分析(B)。A549:未转染组; CtrlsiRNA:转染阴性对照siRNA组; EGF:EGF刺激组; EGF+CtrlsiRNA:转染阴性对照siRNA加EGF刺激组; EGF+siRNA:转染STAT5 siRNA加EGF刺激组; STAT5 siRNA:转染STAT5 siRNA组。 Western blot analysis of STAT5 expression (A) and electrophoresis analysis of COX-2 expression (B) in A549 cells transfected with STAT5 siRNA together with or without EGF stimulation.A549:untransfected; CtrlsiRNA:transfected with control siRNA; EGF:stimulation with EGF; EGF+CtrlsiRNA:transfected with control siRNA and stimulation with EGF; EGF+STAT5 siRNA:transfected with STAT5 siRNA and stimulation with EGF; STAT5 siRNA:transfected with STAT5siRNA.

### 2.3 野生型STAT5及STAT5显性负突变体对A549细胞中STAT5、p-STAT5蛋白及COX-2 mRNA表达的影响

Western blot检测结果表明, 与未处理组的细胞相比, 转染野生型STAT5细胞中STAT5蛋白表达水平明显升高(*P* < 0.05), 而对p-STAT5蛋白表达无明显影响。转染STAT5显性负突变体细胞对STAT5表达无明显影响, 但Western blot可见突变体条带, 而且, 转染STAT5显性负突变体后, EGF对STAT5的活化作用也被明显削弱(*P* < 0.05, 图 5A, 图 5B), 提示STAT5显性负突变体有阻断STAT5激活的作用。另一方面, 与未转染的细胞相比, 转染野生型STAT5细胞中COX-2 mRNA的表达明显升高(*P* < 0.05), 提示非磷酸化的STAT5在A549细胞中有调节COX-2表达的作用。转染STAT5显性负突变体细胞中, EGF上调COX-2 mRNA的作用被明显削弱(*P* < 0.05, 图 5C), 提示p-STAT5在A549细胞中同样具有调节COX-2表达的功能。

**5:**
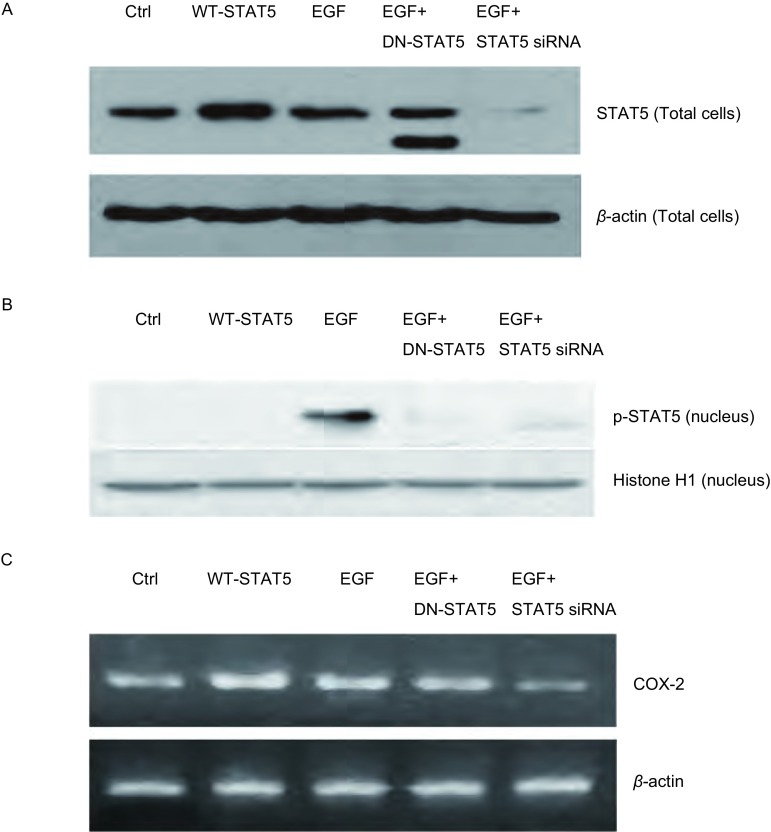
A549细胞转染野生型STAT5, STAT5显性负突变体和STAT5 siRNA后以及受到EGF刺激后对STAT5(A)、p-STAT5(B)表达影响的免疫印迹分析和COX-2 mRNA表达影响的电泳分析(C)。Ctrl:未转染组; WT-STAT5:转染野生型STAT5组; EGF:EGF刺激组; EGF+DN-STAT5:转染STAT5显性负突变体加EGF刺激组; EGF+STAT5 siRNA:转染STAT5 siRNA组加EGF刺激组。 Western blot analysis of STAT5 (A), p-STAT5 (B) and electrophoresis analysis of COX-2 (C) expression in A549 cells transfected with WT-STAT5, DN-STAT5 and STAT5 siRNA together with or without EGF stimulation.Ctrl, transfected with control adenovirus; WT-STAT5, transfected with wild-type STAT5;EGF, stimulation with EGF; EGF+DN-STAT5, transfected with dominant negative STAT5 and stimulation with EGF; EGF+STAT5 siRNA, transfected with STAT5 siRNA and stimulation with EGF.

### 2.4 A549细胞中STAT5表达的变化和STAT5活化对DNA结合活性的影响

转录因子结合实验结果显示, 与未处理组的细胞相比, EGF刺激后的细胞的STAT5 DNA结合活性明显升高(*P* < 0.05)。转染野生型STAT5的细胞未发现STAT5 DNA结合活性发生改变。而转染STAT5显性负突变体和STAT5 siRNA的细胞中, EGF对STAT5 DNA结合活性的刺激作用被抑制(图 6)。提示STAT5显性负突变体和STAT5 siRNA有抑制STAT5 DNA结合活性的作用, 从而影响STAT5的转录活性。

**6:**
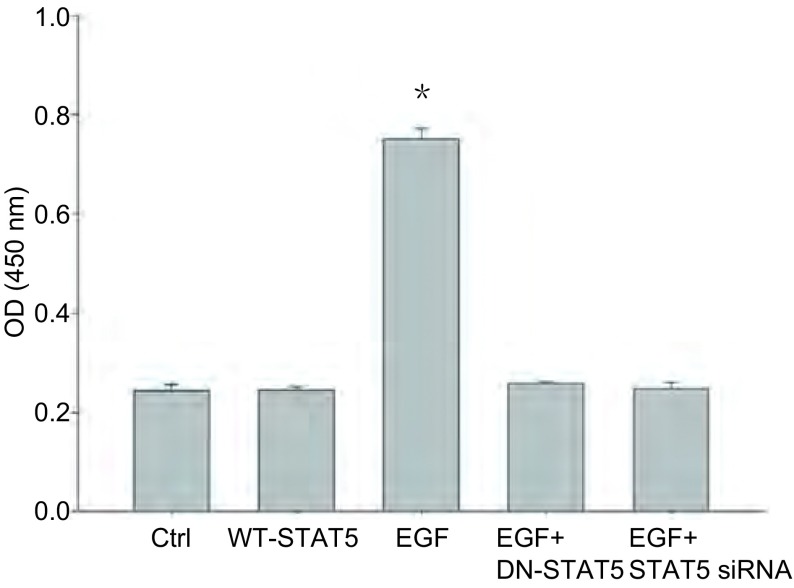
A549细胞转染野生型*STAT5*, STAT5显性负突变体和STAT5 siRNA后以及受到EGF刺激后对STAT5 DNA结合力表达影响的分析。与其它组相比, ^*^P < 0.05, *n*=3。 STAT5 DNA binding assay in A549 cells nuclei transfected with WT-STAT5, DN-STAT5 and STAT5 siRNA with or without EGF stimulation.Data are means± SEM.^*^P < 0.05 when compared with the other four groups.*n*=3.

## 3 讨论

自1992年Fu发现了信号转导和转录激活因子以来, 国内外医学工作者对STAT家族进行了大量研究, 证实STAT家族成员参与了多种细胞因子、生长因子的信号转导, 并调节人体免疫反应、炎症反应和细胞的生长、分化等。研究显示, 在许多恶性肿瘤中, 包括白血病^[6]^、乳腺癌^[7]^、前列腺癌^[8]^、头颈癌^[9]^及NSCLC^[10]^中均存在STAT的异常激活。STAT激活后发生磷酸化, 形成二聚体并穿梭入细胞核, 入核后的STAT与同源的DNA结合区域相结合诱导转录激活^[11]^。在NSCLC中已被证实存在STAT酪氨酸磷酸化现象^[12]^。

在STAT家族的7个成员中, 目前研究最为深入和广泛的是STAT3和STAT5。STAT5分为STAT5a和STAT5b两种亚型, STAT5位于细胞浆内, 受细胞因子或生长因子刺激, 在酪氨酸激酶, 特别是JAK激酶的作用下, 其羧基末端结构域的酪氨酸残基发生磷酸化, 从而使STAT5发生活化^[13]^。磷酸化的STAT5形成同源或异源的二聚体, 穿梭入细胞核内, 识别并结合到靶基因特异启动子的反应元件中^[14]^。

RNA干扰(RNA interference, RNAi)技术是用来研究基因功能的最常用工具, 属于转录后基因沉默(post-transcriptonal gene silencing, PTGS)。而显性负突变体是通过显性负性作用而产生负性调节效应, 在蛋白水平起竞争性抑制作用, 对目的基因表达无影响, 能够和正常受体竞争结合配体, 但是显性负突变体无信号传导功能, 因此对该受体功能起到抑制作用, 并对该受体的表达未造成影响, 而siRNA则会导致该受体表达水平下降。因此, siRNA和显性负突变体是在不同水平起作用的。STAT5是通过磷酸化及去磷酸化来实现其作用的, 磷酸化后生成的蛋白量很少, 若仅用siRNA来抑制目的基因表达并不能完全阐释STAT5的功能。而用显性负突变体则可以代替功能蛋白来研究其缺失后状态, 并对研究人类癌细胞的基因功能非常有用。STAT5显性负突变体的特点是使激活域的C末端的部分或完全失活。Stat5a△740是表达负显性STAT5的质粒, 保留了二聚体形成及DNA结合区域中保守的酪氨酸残基, 可对STAT5a和STAT5b介导的转录产生干扰^[15]^。我们通过对A549细胞进行STAT5显性负突变体转染, 使细胞过表达显性负STAT5, 从而减弱DNA结合活性, 抑制EGF介导的DNA结合活性的增加, 以此研究STA5活化后对COX-2的调节作用。通过对细胞进行野生型STAT5的转染可以使STAT5的表达升高, 借助此方法, 我们可以更深一步地为非磷酸化STAT5对COX-2的调控作用进行阐释。

本研究中, 通过对A549细胞进行EGF的刺激, STAT5 siRNA、野生型STAT5及STAT5显性负突变体的转染, 从多层次多角度探讨了STAT5对COX-2的调控作用, 从而得出如下结论:EGF的刺激可诱导A549细胞中STAT5的活化, 使COX-2 mRNA的表达明显升高, 但STAT5的表达未增加; STAT5 siRNA可抑制STAT5蛋白及COX-2 mRNA的表达, 并可削减EGF对STAT5的激活效应; 野生型STAT5可使STAT5蛋白和COX-2 mRNA的表达升高, 但不能使STAT5发生活化; STAT5显性负突变体可削减EGF对STAT5的活化作用和EGF上调COX-2 mRNA的作用。这些结果提示, 在肺腺癌A549细胞中, STAT5的激活参与了COX-2的调控, 而且是COX-2上调表达的必须条件。另一方面, 非磷酸化的STAT5的表达也是COX-2表达的必要条件, 其可能通过不依赖于磷酸化和转录激活途径来实现其调控作用。COX-2表达很可能受非磷酸化STAT5及磷酸化STAT5双途径的调控。据文献^[16]^报道, STAT5可被EGFR家族激酶激活, 这也与我们得出的STAT5可以通过EGFR信号通路发生活化这一结论相符。

综上所述, 通过我们研究, 有如下新发现:①在体外A549细胞中STAT5无激活; ②EGF能够诱导STAT5的激活, 促使磷酸化的STAT5穿梭入核; ③STAT5的激活是EGF诱导COX-2上调表达的必要条件; ④非磷酸化状态的STAT5可能通过非转录激活的途径参与了COX-2表达的调控。更令我们感兴趣的是在研究中发现非磷酸化STAT5也可能参与了COX-2表达的调控, 还有一个尚未明确的信号通路需要我们继续去探求。我们可以利用STAT5通过磷酸化及非磷酸化双途径来实现对COX-2的调控(图 7)这一特点, 探求一条以STAT5为靶点治疗肺腺癌的新途径, 为更多肺腺癌患者的治疗寻求到新出路。

**7:**
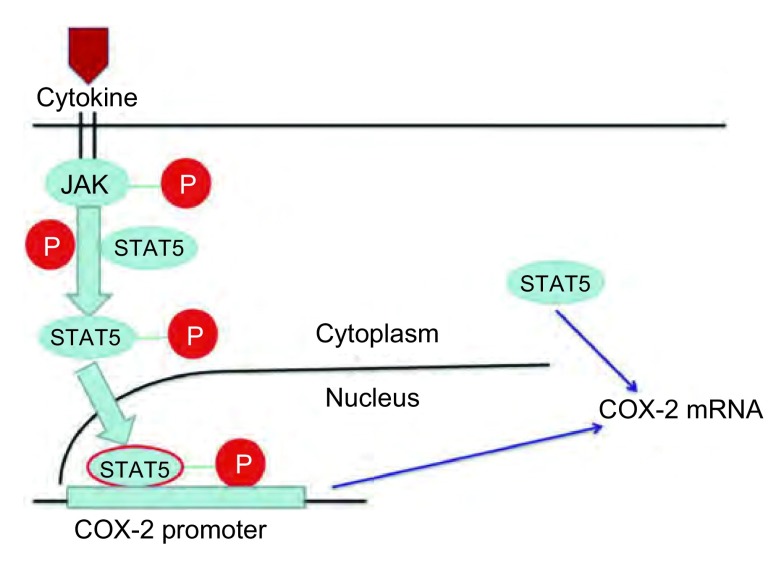
STAT5对COX-2可能存在的两种调控机制。STAT5通过磷酸化及非磷酸化双途径来实现对COX-2的调控。 Schematic depiction of two different potential mechanisms of regulation.STAT5 regulates COX-2 by pathways dependent of phosphorylation and unphosphorylation.
